# Who is paid in pay-for-performance? Inequalities in the distribution of financial bonuses amongst health centres in Zimbabwe

**DOI:** 10.1093/heapol/czab154

**Published:** 2022-01-29

**Authors:** Roxanne Kovacs, Garrett W Brown, Artwell Kadungure, Søren R Kristensen, Gwati Gwati, Laura Anselmi, Nicholas Midzi, Josephine Borghi

**Affiliations:** Department of Global Health and Development, Faculty of Public Health and Policy, London School of Hygiene and Tropical Medicine, 15-17 Tavistock Place, London WC1H 9SH, UK; School of Politics and International Studies (POLIS), University of Leeds, Woodhouse Leeds LS2 9JT, UK; Training and Research Support Centre (TARSC), Harare, Zimbabwe; Danish Centre for Health Economics University of Southern Denmark, 5000 Odense C Denmark & Imperial College London, Faculty of Medicine, Institute of Global Health Innovation, London SW7 2AZ, UK; Ministry of Health and Child Care, Harare, Zimbabwe; Division of Population Health, Health Services Research & Primary Care, The University of Manchester, Manchester, M13 9NT, UK; National Institute of Health Research, Ministry of Health and Child Care, Harare, Zimbabwe; Department of Global Health and Development, Faculty of Public Health and Policy, London School of Hygiene and Tropical Medicine, 15-17 Tavistock Place, London WC1H 9SH, UK

**Keywords:** Health financing, pay-for-performance, inequality, Zimbabwe

## Abstract

Although pay-for-performance (P4P) schemes have been implemented across low- and middle-income countries (LMICs), little is known about their distributional consequences. A key concern is that financial bonuses are primarily captured by providers who are already better able to perform (for example, those in wealthier areas), P4P could exacerbate existing inequalities within the health system. We examine inequalities in the distribution of pay-outs in Zimbabwe’s national P4P scheme (2014–2016) using quantitative data on bonus payments and facility characteristics and findings from a thematic policy review and 28 semi-structured interviews with stakeholders at all system levels. We found that in Zimbabwe, facilities with better baseline access to guidelines, more staff, higher consultation volumes and wealthier and less remote target populations earned significantly higher P4P bonuses throughout the programme. For instance, facilities that were 1 SD above the mean in terms of access to guidelines, earned 90 USD more per quarter than those that were 1 SD below the mean. Differences in bonus pay-outs for facilities that were 1 SD above and below the mean in terms of the number of staff and consultation volumes are even more pronounced at 348 USD and 445 USD per quarter. Similarly, facilities with villages in the poorest wealth quintile in their vicinity earned less than all others—and 752 USD less per quarter than those serving villages in the richest quintile. Qualitative data confirm these findings. Respondents identified facility baseline structural quality, leadership, catchment population size and remoteness as affecting performance in the scheme. Unequal distribution of P4P pay-outs was identified as having negative consequences on staff retention, absenteeism and motivation. Based on our findings and previous work, we provide some guidance to policymakers on how to design more equitable P4P schemes.

**Key messages**

- Although pay-for-performance (P4P) schemes have been implemented across low- and middle-income countries, little is known about their distributional consequences
- We examine inequalities in the distribution of pay-outs in Zimbabwe’s national P4P scheme
- We find that facilities with better baseline access to guidelines, more staff, higher consultation volumes and wealthier and less remote target populations earn significantly higher P4P bonuses throughout the programme
- Unequal distribution of P4P pay-outs was identified as having negative consequences on staff retention, absenteeism and motivation

## Introduction

Contracts that link the remuneration of healthcare workers to pre-specified performance targets have been implemented in over 50 low- and middle-income countries (LMICs) with the hope of improving the quality of healthcare services and health outcomes (Witter *et al.*, 2012; Kovacs *et al.*, 2020). There is a large literature on the impact of such pay-for-performance (P4P) schemes (Basinga *et al.*, 2011; Witter *et al.*, 2012; Eijkenaar *et al.*, 2013; Engineer *et al.*, 2016; Antony *et al.*, 2017; Diaconu *et al.*, 2021). However, previous works have primarily focused on estimating the average effects of interventions (Doran *et al.*, 2008; Basinga *et al.*, 2011; Witter *et al.*, 2012; Diaconu *et al.*, 2021). Consequently, little is known about the distributional consequences of P4P, in particular, relating to the distribution of P4P bonuses themselves.

Distributional effects are relevant in P4P, as incentivized agents (healthcare providers or facilities) with different characteristics likely differ in their ability to provide health services and therefore also differ in their ability to respond to predetermined performance targets. It is easy to imagine how facilities lacking resources and staff might find it more difficult to meet P4P targets, especially in P4P programs without readiness investments prior to implementation or no built-in facility improvement incentives (Binyaruka and Anselmi (2020). Moreover, incentivized outcomes in P4P schemes are often not fully under providers’ control and depend on local area and population characteristics. For instance, 83% of P4P schemes in LMICs incentivize consultation volumes (Kovacs *et al.*, 2020)—which are in part determined by population demand for healthcare services and households’ ability to pay. Generally speaking, if disadvantaged facilities were best able to respond to P4P incentives, then P4P payments would reduce existing inequalities in healthcare by providing additional resources to these facilities. However, if financial bonuses are primarily captured by those who are already better able to perform, then P4P payments could widen and reinforce existing inequalities between healthcare facilities. A few studies in the USA have investigated the characteristics of hospitals that perform poorly in a P4P scheme that involved penalties for readmission and found that smaller hospitals, teaching hospitals and those managing more complex patient populations were penalized more frequently (Joynt and Jha, 2013; Rajaram *et al.*, 2015; Horwitz *et al.*, 2018). In LMICs, a dedicated study was conducted in Tanzania, which examined the association between P4P pay-outs and facility characteristics in a pilot programme implemented in one region of the country (Binyaruka *et al.*, 2018). Yet, in general, there remains a limited number of dedicated studies examining P4P pay-outs and facility characteristics in LMIC settings (Binyaruka *et al.*, 2018; Diaconu *et al.*, 2021). This paper adds to this line of research and also reflects on how incentive design may influence inequalities in the distribution of P4P bonuses.

This article examines inequalities in the distribution of performance pay-outs in Zimbabwe’s national P4P scheme (2014–2016), known locally as the Results-Based Financing (RBF) programme, and how these change over time. This is a mixed-methods study that combines quantitative data on bonus payments with findings from semi-structured interviews. We investigate the degree to which facility and local area characteristics at baseline are associated with bonus payments during the RBF programme.

We find that in Zimbabwe, facilities with better *baseline* access to treatment guidelines, more staff, higher consultation volumes and wealthier and less remote catchment populations earn significantly higher bonuses throughout the programme. We do not find evidence to suggest that these inequalities disappeared or became less pronounced over time, corresponding with a Matthew Effect of Accumulated Advantage, which postulates that initial advantages tend to beget further advantages, and disadvantages further disadvantages, among individuals and groups over time. The qualitative data confirmed these findings, as respondents identify facilities’ baseline structural quality, leadership, catchment population size and remoteness as affecting performance. Unequal distribution of P4P bonuses was perceived as having negative consequences for staff retention, absenteeism and motivation. Based on the findings of this study and previous work, we provide design recommendations for more equitable P4P schemes.

## Background

### Study context

Health services are provided by a mix of public facilities (government and local authority facilities), non-profit-run facilities, religious/mission organizations and the private sector (for-profit facilities). However, the public sector is the major provider of health services in Zimbabwe (Table 1). The majority of the population of Zimbabwe obtain health services from government, local authorities and mission facilities, while private for-profit facilities cater for mainly formally employed people on health insurance in mainly urban areas. However, private health insurance coverage remains at low levels: in 2015, 89% of women and 88% of men did not have health insurance (ZIMSTAT), 2015). Out of Pocket (OOP) expenditure is relatively high: in 2010, it was 39% of total health expenditure whilst government funding accounted for 18%, donors 19%, and private companies and others 24% (MoHCC, 2016).

**Table 1.** Distribution of health service facilities by provider and level, 2015

	Number of facilities			
Level	Mission	MoHCC	Council/Local Gvt	Private
Primary care facilities—usually no doctors—only Registered General Nurse(s) and primary care cadres	25	1444	96	69
Secondary care facilities—Approx. two doctors supported by nurses	12	106		32
Tertiary care facilities/Provincial hospitals—few specialist doctors supported by nurses	8			
Quaternary care facilities/Central Hospitals—most specialist doctors stationed here	6			

Source: MoHCC (2016).

### The Zimbabwe RBF programme

The Government of Zimbabwe initiated the RBF programme in September 2010 in collaboration with the World Bank (WB). The programme intended to ‘improve the availability, accessibility and quality of key reproductive and child health services and their optimal utilisation’ (MoHCC, 2011, p. 23) in facilities at all levels (health centres and hospitals) and focused on rural areas (i.e. all provinces except for Harare and Bulawayo). The RBF programme was introduced in three phases: a pilot phase (two districts, July 2011–March 2012), a second phase (16 additional districts, April 2012–March 2014) and a national roll-out (42 remaining rural districts, April 2014 to present). The National Purchasing Agent (NPA)^1^ for the 18 districts that joined in the pilot and first phase of the programme was Cordaid. The NPA for the 42 districts that joined as part of the national rollout in 2014 was Crown Agents. Both NPAs are governed by a steering committee chaired by the Ministry of Health (Brown *et al.*, 2018).

Table 2 summarizes the design of the RBF programme and its financial incentive structure in phase three for rural health centres, based on the conceptual framework developed by Kovacs *et al.* (2020). The financial bonus that facilities receive has three components: payment for the quantity of services (quantity bonus), payment for the quality of services (quality bonus) and a remoteness bonus (The World Bank, 2016; Brown *et al.*, 2018). The quantity bonus is based on unit prices for 16 indicators (see Appendix A1)—e.g. 0.10 USD are paid for each new out-patient consultation. The quality bonus is based on a percentage score on a quality checklist, completed every 3 months during facilities visits, which captures structural and process quality of care, management quality and user satisfaction. The amount paid for the quality of care bonus is calculated as a proportion of the quantity bonus.^2^ The remoteness bonus is based on an assessment of distances to referral facilities, availability of communication, road access and population size. Each of these dimensions is weighted and the maximum amount that can be earned for remoteness is 30% of the quantity amount (see Appendix A1 for details).

**Table 2.** Incentive design in Zimbabwe’s national RBF programme, for rural health centres (2014–2016)

Measures of performance incentivized
- *Healthcare visits (number of out-patient consultations)* - *Process quality of care (first ANC visit completed on time, growth monitoring of children under five)* - *Structural quality of care (drugs and equipment, infection control)* - *Management practices (stock management, health information system management)* - *User satisfaction*
Whose performance is measured and who (ultimately) receives the payment?
- *Rural health centres*
Payment attributes
- *Quarterly payments of 1694 USD per facility on average, with penalties for misreporting* - *Time-lag between reporting performance and receiving payment is 4 months* - *Coupling of bonus and salary payments depends on the facility* - *25% of bonus can be spent on staff bonuses and 75% needs to be reinvested in the facility (infrastructure, supplies, equipment)*
Basis for payment
- *Per action (quantity bonus) as well as thresholds of performance (quality bonus)* - *Ranking based on own performance* - *Payment adjustment based on remoteness*
Gaming safeguards
- *Data verification by communities, district, regional and national managers* - *Bonus withheld if reported outcomes off by more than 5%*
Implementation, technical assistance, complementary reforms
- *Funded by pooled multi-donor Health Development Fund from 2014* - *Implemented by Crown Agents (42 districts) and Cordaid (18 districts)* - *Strengthened implementation of the governments’ 2010 user-free removal law*

#### Conceptual framework

The relationship between P4P pay-outs and facility and local area characteristics is not straightforward conceptually. On the one hand, it is possible that facilities with better baseline structural quality (more guidelines, better access to drugs), more staff, higher consultation volumes and wealthier and less remote target populations are better able to perform and therefore earn higher bonuses. This would be in line with a Matthew Effect of Accumulated Advantage (Rigney, 2010), where groups that are initially (dis)advantaged become even more (dis)advantaged over time, creating widening gaps between those who have more (and earn more) and those who have less (and earn less). The expectation under the Matthew Effect is that facilities with higher baseline structural quality would see higher increases in performance and bonuses over time relative to facilities starting with lower structural quality.

On the other hand, it is possible that facilities that start from a lower level (say with lower consultation volumes) find it easier to increase both the quantity and quality of services provided. This could be because, in facilities with lower baseline performance, there are potentially more ‘low-hanging fruit’ for improving performance—for instance, changes in management or outreach processes that are not costly. Facilities that already perform at a high level at baseline might find it difficult or costly to further increase the quantity or quality of services provided. One concern is that initial increases in performance from facilities with a lower baseline level might be difficult to maintain and might flatline or even decrease over time, as they are unable to up-keep momentum (Kuunibe *et al.*, 2020). Moreover, the P4P scheme in Zimbabwe included a remoteness bonus, which in theory should provide facilities with more remote target populations with additional payments. This could allow them to ‘level up’ and address historical hindrances responsible for underperformance.

## Methods

This is a mixed-methods study that combined quantitative data on P4P bonuses (2014–2016) with 28 multi-stakeholder interviews (conducted in 2017) and a desk review of published and grey literature. Ethical approval was received from the authors’ institutes.

### Quantitative methods

#### Data

The quantitative analysis relied on three main sources of data. First, we used data on quarterly (USD) pay-outs made to facilities in the RBF programme, provided by Crown Agents. These data contained information on the 42 districts managed by Crown Agents (817 primary healthcare facilities) in phase three of the programme (2014–2016)^3^. All districts were newly exposed to the intervention in 2014. Pay-outs were adjusted for inflation and expressed in 2015 USD.

Second, we used baseline data from an impact evaluation of the RBF programme (The World Bank., 2016). These data were collected by the World Bank between December 2011—February 2012 in 197 facilities and provide information on healthcare facility characteristics prior to the implementation of the programme (GIS-location, infrastructure, availability of treatment guidelines, drugs and equipment, staffing levels, consultation volumes). They also linked each health facility to its nearest cluster of households that participated in the 2010 Zimbabwe Demographic and Health Survey (DHS).^4^ Third, data from the 2010 DHS were used to approximate the socio-economic status of the local area in which facilities are located. We used these data to establish the facility and local area characteristics before the start of the P4P programme.

**Figure 1.**
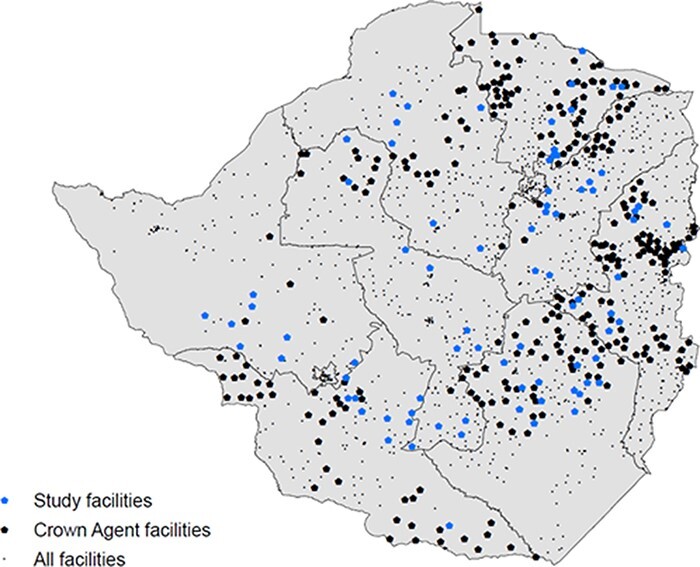
Study facilities

The quantitative analysis focused on a sample of 87 primary health facilities (rural health centres) for which data on P4P bonus payments as well as facility and local area characteristics are available. In the study period, Crown Agents managed 179 rural health centres, meaning that our sample represents 48% of these facilities. Figure 1 shows the location of the facilities included in this study, other facilities managed by Crown Agents and all other facilities in the country for which GIS data were available.^5^

#### Outcomes

The quantitative analysis is descriptive and focuses on whether facility and local area characteristics before the start of P4P incentives are associated with the size of bonus payments received during the programme. The aim of the paper is not to make causal claims about the effect of P4P on outcomes, but to examine the distribution of financial bonuses.

In terms of facility characteristics, we captured the availability of treatment guidelines (proportion of 22 guidelines relevant to the provision of primary care available), the number of clinical staff working in the facility (doctors, nurses and midwives), consultation volumes (the total number of outpatient consultations conducted in the past month) and availability of drugs (proportion of 43 drugs essential to the provision of primary care available in the facility). To capture the characteristics of the area in which facilities are located we approximated the level of wealth of households as well as remoteness. We used the cluster-average DHS wealth index of the DHS cluster nearest to the health facility as a proxy for the wealth of the target population. The DHS wealth index is a composite measure of households’ living standards and captures access to assets, materials used for house construction and the type of water and sanitation access. We proxied remoteness as the kilometre distance between each health facility and the provincial capital as well as the nearest district hospital. Remoteness (in reference to provincial capitals and hospitals) is captured in terms of quintiles—as all health facilities in the sample are divided into five equally sized groups, where the lowest quintile (Q1) is the one furthest from provincial capitals and hospitals. Local-area wealth is also measured in quintiles. All DHS clusters in the sample are divided into five equally sized groups, where the lowest quintile (Q1) is the least wealthy—meaning that wealth is relative to clusters in the sample, rather than the country as a whole.

#### Data analysis

To determine whether facility and local area characteristics at baseline are associated with subsequent receipt of P4P bonus payments, we used the following time-series linear regressions:

(1) $${Y_{it}} = {\beta _0} + {\beta _1}Characterstic{s_i} + {\delta _1}{Q_t} + {\beta _3}\textrm{province} + {\varepsilon _{it}}$$

Where ${Y_{it}}$ is the logged inflation-adjusted P4P bonus received per facility per quarter (in 2015 USD). ${Characterstics_{i}}$ refers to facility and local-area characteristics at baseline (shown in Table 2). All models control for the time in quarters $\left( {{Q_t}} \right)$ and province fixed effects. For ease of interpretation, we show the predicted P4P bonus (in USD), based on whether facilities are 1 SD above or below the mean for each variable. Predicted values were calculated based on time-series linear regressions as specified in equation 1 above.

To examine how the associations between the facility and local area characteristics and bonus payments change over time, we included an interaction term, such that:

(2) $$\eqalign{ & {Y_{it}} = {\mkern 1mu} \,{\beta _0} + {\beta _1}\;Characterstic{s_i} + {\beta _2}\;Characterstic{s_i}{\rm{*Year}} \cr & \,\,\,\,\,\,\,\,\,\,\,\,\,\,\,\,\, + {\delta _1}{Q_t} + {\delta _2}{\rm{Year}} + {\beta _3}{\rm{province}} + {\varepsilon _{it}} \cr} $$

We interacted with each variable capturing facility and local area characteristics with the year in which the bonus was earned.

#### Qualitative methods

We conducted 28 semi-structured interviews (lasting approximately 1 h) with a cross-section of Zimbabwe RBF stakeholders, including officials from the Ministry of Health and Child Care at national (*n* = 4), provincial (*n* = 5), district (*n* = 4) and facility levels (*n* = 2), the two NPAs (*n* = 6), World Bank and Health Development Fund (HDF) external financer (*n* = 2), civil society organisations (*n* = 3), independent ‘counter-verifiers’ that checked internal evaluation accuracy in Zimbabwe (*n* = 1) and a UN agency (*n* = 1). Out of all the respondents, 15 respondents were male and 13 were female. Stakeholders were identified via a stakeholder assessment conducted during a desk review involving an extensive online search for documents about the RBF programme in Zimbabwe (see Appendix A2 for how the review was conducted). The assessment generated a master list of all programme stakeholders, their affiliations, roles and geographic locations. The key informant list was assessed by four members of the research team and a priority list of 35 interviewees was produced to capture a representational cross-section of the programme including geographical location of facilities (Kadungure, Brown, Loewenson and Gwati). The loss of seven interviewees from the original list of 35 was a result of changed employment or unavailability during the interview period.

The interview guide contained a single structured question to investigate why facilities in some areas performed better than others, namely: ‘Does the Results Based Financing programme work better in some areas or facilities than others?’. The question was deliberatively left open-ended so as to not bias response and to allow information to emerge inductively. The question was then followed up using semi-structured interviewing techniques to elicit additional responses identifying why respondents believed the programme performed better in some settings, which facility and contextual characteristics affected performance, and with what effects.

Except for two interviews conducted by phone, all interviews were conducted by one researcher at the stakeholder’s location of choice in Zimbabwe. All interviews were conducted in English and were recorded, with transcription taking place as close to the interview date as possible. The original recordings and corresponding transcripts were immediately uploaded onto the secure drive of the author’s institute. The interviews were reviewed on an ongoing basis by two additional members of the research team and continued until there was consensus that data saturation had been reached.

Thematic analysis was conducted by three members of the research team with inter-rater agreement determined by consensus. We inductively grouped interview responses into themes: the types of inequity perceived between facilities; explanations for why those inequities existed (before and after the programme); facility and contextual characteristics affecting performance, perceptions of how P4P processes mitigated or enhanced identified inequities and the consequences for a facility or personal performance. The findings from the qualitative analysis were validated and enhanced through presentation and discussion at two national workshops in Zimbabwe attended by 77 key country stakeholders across all programme levels, including staff from high and low performing facilities and from remote and urban settings (MoHCC, NIHR and TARSC, 2020).

## Results

### Sample description

Table 3 shows facility and local area characteristics for the 87 rural health centres that are included in the quantitative analysis. Although our sample is composed of rural health centres, which are intended to serve the same number of people, there is substantial variation in facility characteristics—in particular in terms of the number of staff and baseline consultation volumes.

**Table 3.** Facility and local area characteristics at baseline (*N* = 87)

	Mean	SD	Min	Max
Proportion of clinical guidelines available	0.81	0.13	0.50	1.00
Number of clinical staff	3.71	3.62	1.00	19.00
Total monthly out-patient consultations	799.53	932.95	148.00	7909.00
Drug availability index	0.60	0.07	0.40	0.95
Proportion in two lowest wealth quintiles (*N* = 56)	0.54	0.50	0.00	1.00
Distance to provincial capital (km) (*N* = 82)	85.05	45.29	9.59	240.42
Distance to nearest district hospital (km) (*N* = 82)	41.65	23.88	2.91	112.23

**Figure 2.**
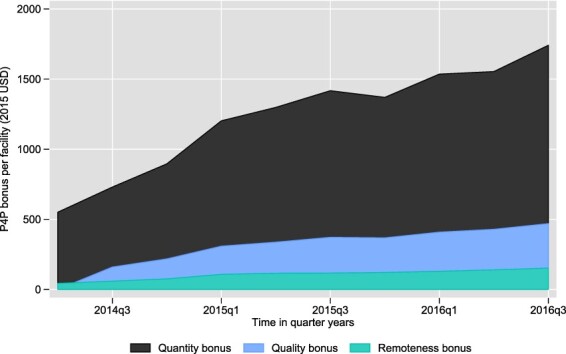
Bonuses paid per facility per quarter

Figure 2 shows the average quarterly P4P bonus received by facilities. The amount of financing that facilities receive increases over time (i.e. quarters)—from 603 USD in Q2 2014 to 1779 USD in Q2 2015, to 2341 in Q2 2016. The quantity bonus makes up 75% of the total bonus on average, compared to 19% for the quality bonus and 6% for the remoteness bonus.

### Quantitative analysis

**Figure 3.**
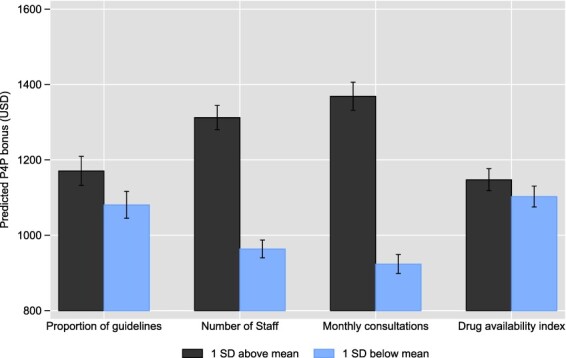
Facility characteristics at baseline and predicted P4P bonus (based on time-series regressions shown in Table A1)

We found that facilities with better access to clinical guidelines, more staff and higher consultation volumes before the start of the intervention are able to earn higher P4P pay-outs (Figure 3, based on results of regressions presented in Table A1). Facilities that were 1 SD above the mean in terms of access to guidelines, earned 90 USD more per quarter, than those that were 1 SD below the mean on this variable. Those that were 1 SD above the mean in terms of the number of staff earned 348 USD more per quarter. Facilities that were 1 SD above the mean in terms of consultation volumes earned 445 USD more per quarter. We did not find a significant association between the availability of drugs at baseline and subsequent P4P pay-outs.

**Figure 4.**
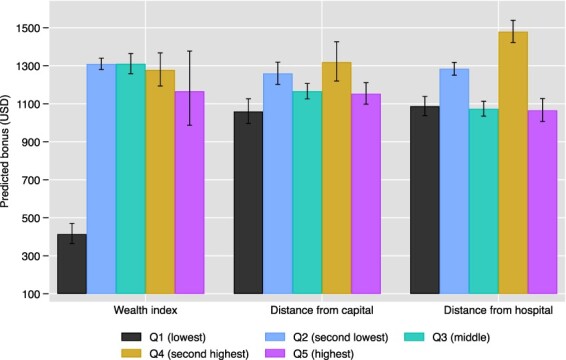
Local area characteristics at baseline and predicted P4P bonus (based on time-series regressions shown in Table A2)

Note: Q1 refers to villages with the lowest level of wealth and facilities with the highest remoteness (i.e. largest distance to provincial capitals and hospitals).

We found some evidence that facilities located in areas where households are wealthier, as well as those that are closer to provincial capitals and district hospitals, earn higher P4P bonuses (Figure 4, based on results of regressions presented in Table A2). Facilities with DHS villages in the poorest wealth quintile (Q1) in their vicinity earned less than all others—and 752 USD less per quarter than those with villages in the richest quintile (Q5). However, we found no significant differences in bonus payments between other wealth quintiles. Similarly, facilities that were furthest from provincial capitals (those in Q1) earned less than all others, and 94 USD less per quarter than those in the least remote quintile (Q5). When remoteness was measured in terms of distances to district hospitals results were more mixed, as facilities in the second least remote quintile (Q4) earned the highest pay-outs (392 USD more than the least remote facilities).

Table A3 in the Appendix includes facility and local-area characteristics within a single model. This is not done in the main specification as several of the variables of interest are highly correlated and potentially constitute bad controls (Cinelli *et al.*, 2021). Results are qualitatively very similar when these factors are included not individually but alongside one another. We find that availability of guidelines, the number of staff, as well as total consultation volumes, are quite consistently associated with higher bonus pay-outs (Models 1–4 in Table A3). We also find that the availability of drugs is significantly associated with higher bonus pay-outs when the local-area socio-economic status is controlled for (Model 2). As shown in Model 2, the socio-economic status of the local area continues to be relevant for bonus pay-outs, as facilities with poorer villages in close vicinity appear to earn lower bonuses. Results on remoteness (Models 3–4 in Table A3), do not show a clear pattern when facility characteristics are included in the same model—potentially due to the high degree of correlation between these variables.

### Differences by bonus component

Results for the quantity and quality component of the bonus are highly similar to those shown above (Tables A4–A7), as better equipped facilities as well as those located in wealthier or less-remote areas earned higher quantity and quality pay-outs. This is not the case for the remoteness bonus. We find that facilities with better availability of drugs prior to the start of the intervention earned smaller remoteness bonuses (Table A8). Facilities that are further from the provincial capital generally earned higher remoteness bonuses (Table A9). Contrary to what one might have expected (as the remoteness bonus was supposed to make up for facilities being located in more rural areas), facilities that were closer to the nearest district hospital earned higher remoteness bonuses (Table A9).

### Time trends

We did not find evidence to suggest that the magnitude of the association between the P4P bonus and facility and local area characteristics at baseline changed over time (Tables A10 and A12). This is also the case when the facility and local-area characteristics are included in the same model (Tables A11 and A13).

#### Qualitative analysis

This section presents results from the qualitative analysis.

### Facility characteristics and performance

Although a number of interviewees suggested that there was no discernible difference between areas or facilities (*n* = 7), a large majority of interviewees (*n* = 21) believed that the RBF programme worked better in some facilities than others. These findings were verified and further elaborated on by the 77 stakeholders who attended the two national workshops.

Respondents identified some facility characteristics as being critical to performance and the ability to earn bonuses. Facilities facing staff shortages, or lacking trained staff, were close to uniformly reported to suffer from underperformance and reduced bonuses (*n* = 25). As one district nursing officer suggested, ‘*some indicators fail because there are too many tasks to be done. We are understaffed and without capacities to perform well, which causes burnout. Whilst pressure of work may motivate others to work better, others become uncooperative and start to have bad attitudes.’* Respondents reported that facilities with larger consultation numbers also received higher bonuses than facilities in areas with less demand (*n* = 28).

### Local area characteristics and performance

Some of the qualitative interviews highlighted the interplay between local area characteristics such as remoteness and catchment population size, and facility characteristics or consultation volumes (*n* = 11). For example, a Crown Agents programme manager claimed that ‘*when a facility has a small catchment area, it reaches a plateau and cannot attract more patients beyond a certain point, so it loses out on revenue compared to larger catchment area facilities. Low catchment areas needed some input-based financing first, then RBF [Results Based Financing]’*. Such concerns were echoed by facility staff. For example, a nurse in a rural clinic responded that performance and bonuses *‘depends on catchment area, ours is small and out of the way, only about 6000 people. Works better in areas with large catchment population’*.

Respondents associated the inequity in bonus payments with the design of the quantity component of the incentive scheme, which is based on consultation volumes. The remoteness bonus was seen as insufficient to offset this. For example, a national level stakeholder claimed ‘*we have suspected that low catchment areas have not done as well under the programme as more populated areas. Since there is no cap on RBF quantity, the number of patients served will increase bonuses, which increases motivation. The equity bonus was meant to compensate for this, but there are signs that it is not enough and that morale and performance have been affected*’.

A number of respondents argued that remote sites often suffered from demand-side problems that reduced the volume of consultations, including a lack of reliable transportation or a difficult terrain complicating access (*n* = 7). As one respondent suggested, *‘sometimes when it rains it is very hard for patients to get to us and transportation is not always reliable, so we miss potential bonuses’*. Demand-side challenges were noted to be a problem across remote sites. As noted by a national level stakeholder, *‘Health facilities had to come up with creative strategies to reach out all target groups, but demand-side efforts weren’t built into the programme and created difficulties for some remote facilities’*.

Remoteness also caused problems on the supply side, in terms of supervision and facility maintenance. As noted by a Crown Agents team leader, *‘travel issues hinder some*  *districts. Repairs are expensive and earnings are not enough. During programme design, vehicles were part of the programme, but somewhere along the line, the monies for vehicles were used for something else. RBF money should be used for vehicle maintenance so that they can be used for supervision and other functions to boost performance’*.

The interviews also reflected a general perception that facilities in wealthier areas performed better, as they were better able to attract more highly trained staff who were able to follow the reporting guidelines, although this was not consistent across all facilities (*n* = 16). As one Cordaid programme manager argued, *‘there is also a widely held belief that RBF works better in elite areas, which may be down to better facilities with more properly trained staff and reporting, yet I think you’ll find pockets of poor performance in wealthier areas too’*.

### Leadership

Some interview respondents (*n* = 6) and workshop participants believed that clinic leadership and the personal motivation of staff members most determined whether a facility performed better and thus received higher bonuses. As one provincial level respondent suggested, *‘I hear that catchment population may matter to some extent, but I think it largely depends on the institution management, not geography’*. Another respondent suggested that facilities in wealthier areas could attract better leadership, with more trained staff, and more resources at their disposal, *‘allowing for success’* also suggesting that remote sites were less attractive to health professionals than suburban locations.

A number of respondents suggested that some districts performed better than others due to governing bodies that had a higher level of acceptance of the programme, stronger P4P championing and better supervision (*n* = 16). For example, a respondent from CordAid suggested that *‘it comes back to the human factor. There are districts were everything works. It comes back to leadership and motivation at district and provincial level, which translate to stronger and better performing facilities’*. When asked about protocol adherence, a Crown Agents programme manager suggested that this was again due to district leadership and supervision, claiming that *‘whether or not the P4P protocols were fully in place and used appropriately came down to how the province and district made this happen. They had a decentralised management role and ultimately determined whether facilities were properly trained, supervised and adhering to procedures’*. Again, this raised questions about whether wealthier areas could attract more experienced managers while also having appropriate baseline resources for more successful P4P implementation.

### Consequences of unequal distribution of bonuses

Interviews also allowed us to understand some perceived consequences of the unequal distribution of bonuses amongst facilities.

The perception of existing inequalities in bonus distribution was understood to have impacted staff morale and performance. One member of the Zimbabwe health services board suggested, *‘I assessed adjacent districts, one had happy employees as their catchment area was large and the other not so happy as theirs was smaller. We felt there needed to be a better formula to bring about equity. It’s not the fault of a midwife who slept on duty and only delivered one baby and the other midwife in a different catchment that delivered 10 due to higher volumes’*. Another district nursing officer offered a similar claim, stating *‘I spend the whole night on duty, we were there, and there were no patients coming, why should we be penalized as underperforming?’*

In terms of potential knock-on effects on other goals of the RBF programme such as staff retention in remote areas, one district medical officer argued that *‘RBF works better in high volume sites than low volume sites. In high volume sites, some even earn $6000 per quarter. For most staff the drive with RBF is to get to a high-volume clinic with less staff, where there are few health workers to share the money’*. This incentive to work in high volume areas could explain why remote sites were reported to underperform due to staff shortages, retention problems, and greater absenteeism, which were reported to have undermined performance.

## Discussion

Overall, our quantitative analysis suggests that in Zimbabwe, facilities with better structural quality, more staff and wealthier and less remote target populations at baseline earn higher P4P pay-outs throughout the programme. We do not find evidence to suggest that these inequalities disappeared or become less pronounced over time. Evidence from interviews offers some insights to help confirm and explain these results and identifies other factors that could explain differences in performance between facilities. For example, respondents identified certain facility characteristics such as appropriate human resources, leadership, training and equipment as affecting performance and bonus pay-outs. Respondents also believed that local area characteristics affected facility performance, with those in wealthier areas having distinct advantages such as more and better-trained staff and management, which translated into better ability to perform. Moreover, small catchment size and remoteness (which was often perceived as being synonymous by interviewees) was seen as affecting performance and bonus distribution, since bonuses were tied explicitly to client volume. This underperformance had knock-on effects associated with staff retention, absenteeism, and motivation.

Our findings stand somewhat in contrast to previous evidence from LMICs. A previous study examined how pay-outs in a P4P scheme correlate with facility characteristics (Binyaruka *et al.*, 2018). Using data from facilities in one region in Tanzania, Binyaruka *et al.* (2018) found evidence of an inverse equity hypothesis, where inequalities that arise at the start of the P4P scheme diminish over time. We do not find evidence for such a process in this study, as inequalities at the beginning of the scheme are as pronounced 2 years later. One potential explanation of the difference between Tanzania and Zimbabwe could relate to incentive design. The P4P scheme in Tanzania did not reward absolute service volumes, but improvements relative to a baseline. Hence, even though there was no remoteness bonus built into the scheme in Tanzania, facilities with low average service volumes were not penalized as rewards were calculated in reference to their own baseline performance.

One might be surprised that we find inequalities in the distribution of bonuses in Zimbabwe’s RBF scheme, as the programme appeared to have been designed to combat inequities. Part of the bonus was based on remoteness meaning that facilities in rural areas (i.e. long distances to referral facilities and poor availability of roads) received an additional bonus. Moreover, only rural districts were allowed to take part, as facilities in the richest districts in the country (i.e. Harare and Bulawayo) were not included in the scheme. In addition, in keeping with other LMIC schemes (Binyaruka and Anselmi, 2020), a large share of the bonus was for reinvestment in facility infrastructure, supplies and equipment which would be expected to even out baseline differences between facilities, reducing potential inequities. We sense that a key reason why the P4P scheme in Zimbabwe nonetheless favoured facilities that were better able to perform is the strong emphasis on the number of services delivered. Facilities could earn the greatest share of the bonus by providing a large volume of services. In addition, both the quality and the remoteness bonus were calculated as a proportion of the quantity bonus. Therefore, facilities with low consultation volumes—in areas with low demand for healthcare or low population density—were disadvantaged. Zimbabwe’s national P4P scheme is no exception in this regard. Based on a recent review, 83% of identified P4P schemes in LMICs reward service volumes (Kovacs *et al.*, 2020). For instance, a scheme in Benin incentivized the number of curative consultations done (Antony *et al.*, 2017) and a scheme in Afghanistan incentivized the number of ante-natal and post-natal consultations (Engineer *et al.*, 2016).

The clear advantage of incentivizing service volumes is that these are easy to measure and might create stronger incentives for providers, as each action is associated with a reward (Kovacs *et al.*, 2020). However, this study suggests that such a design could have potentially negative equity implications. In terms of scheme design, there are several alternatives to incentivizing service volumes alone, which could address equity concerns. One option would be to reward *improvements* in the volume of services provided, rather than absolute levels. As noted above, this was the case for the P4P scheme in Tanzania (Pwani) where, for instance, the increase in the number of facility-based deliveries relative to a baseline were rewarded (Binyaruka and Borghi, 2017). Another option would be to create performance groups, based on relevant characteristics such as local area income or population density, and reward service volumes only within these groups. Such an approach was used in Brazil’s national P4P scheme, where municipalities were divided into groups based on socio-economic characteristics and bonus payments were estimated based on the relative performance of facilities within these groups (Kovacs *et al.*, 2021). In Brazil’s national P4P scheme, performance inequalities that existed at baseline disappeared over time—potentially due to its design (Kovacs *et al.*, 2021). A final possibility would be to incentivize a simple measure of service volumes but provide disadvantaged areas with a fixed amount based on relevant characteristics, to create a more level playing field. In Zimbabwe, the remoteness bonus could have fulfilled such a function. However, the amount facilities received for remoteness was calculated as a proportion of what was earned for service volumes (i.e. the quantity bonus), meaning that facilities with low service volumes also received smaller top-ups for remoteness. We find some evidence that the distribution of the remoteness bonus was pro-rich, as facilities with wealthier catchment populations, as well as those located closer to district hospitals were paid more and an inconsistent distribution in terms of distance from the provincial capitals. Our findings suggest that a broader set of criteria may need to be considered to ensure equity in the allocation of bonuses across providers.

We found evidence of ‘within facility’ inequities in the distribution of bonuses in our policy review. The staff incentive payment system was overall seen as rewarding ‘qualification’ rather than ‘workloads’ and was developed using top-down approaches (The World Bank, Cordaid and MoHCC, 2013; The World Bank and MoHCC, 2015). Two cadres stood out as having been worst affected: Primary Care Counsellors (PCCs) and Village Health Workers (VHWs). PCCs were responsible for counselling women seeking MNCH care on HIV/AIDs, a core component of the RBF program, yet their grade was not recognized by the MOHCC and received the lowest incentives despite their significant RBF-related workloads (The World Bank, Cordaid and MoHCC, 2013; The World Bank and MoHCC, 2015). VHWs were responsible for implementing growth monitoring and distribution of commodities and providing services for family planning within the community, yet they did not receive incentives. Perceptions of inequities in staff bonus distribution between high earning and low earning members appeared to be worse in facilities where communication between the nurse in charge and the other workers at the clinic was poorer (The World Bank and MoHCC, 2015). This suggests that there is also a need to look at inequities not only across health facilities but also within.

This study is limited in five main respects. First, due to data availability, the quantitative analysis only focuses on a relatively small sample of Crown Agents facilities over a two-year period. Although it is encouraging that many of the factors that were identified as relevant re-emerged in interviews conducted in a broader population, it is unclear to what degree findings apply beyond the study sample. Second, the quantitative analysis is restricted by limited access to covariates. It would have been interesting to study the role of population density, transportation infrastructure or management quality but these data were unfortunately not available at the local area level. Third, in studying local area characteristics health facilities were linked to the nearest cluster of 25–30 households participating in the DHS. The characteristics of these households are likely only a proxy for the characteristics of facilities’ genuine catchment population. Fourth, the majority of documents reviewed for the qualitative component of the study were obtained from organizations that were implementing the programme, specifically the two NPAs, the MoHCC and the World Bank. Although independent sources were consulted, interviewed and triangulated where possible, most P4P evaluations in Zimbabwe were conducted by implementers, which may increase the potential for bias. Finally, although we targeted a broad cross-section of stakeholders for the interview, complemented by two stakeholder workshops, with response saturation and inter-rater consensus, there is some potential for bias in responses given the modest sample size.

## Conclusion

Based on a sub-sample of facilities, our results suggest that Zimbabwe’s national P4P scheme appears to have primarily rewarded facilities that were already better able to perform at the start of the scheme. These findings can be taken to suggest that the P4P scheme appears to have reinforced pre-existing inequalities and inequities within the health system. These findings are in line with a Matthew Effect, as initial advantages tend to beget further advantages, and disadvantages further disadvantages, creating widening gaps between those who have more (and earn more) and those who have less (and earn less) (Rigney, 2010). Whilst there is little evidence on the topic, previous work on P4P schemes in Brazil and Tanzania do not find evidence for a Matthew Effect. This suggests that P4P design can potentially mitigate this effect or that contextual background conditions can significantly diminish or exacerbate the saliency of this effect in practice.

Despite the widespread implementation of P4P in LMICs, this study is one of the few to investigate the equity implications of such schemes. Further research is needed to inform policy on how to design equitable P4P schemes.

## Supplementary Material

czab154_Supp

## Data Availability

The quantitative data underlying this article were provided by the World Bank and Crown Agents. These data will be shared on request to the corresponding author with permission of World Bank and Crown Agents.

## Endnotes

1. NPAs are contracted third-party implementers and fundholders for health service purchasing as part of P4Ps ‘separation of functions’.

2. For example, facilities scoring 51–60% on the quality checklist receive a quality bonus equal to 15% of their quantity bonus.

3. Hence, we only have data on the 42 districts managed by Crown Agents and lack information on pay-outs made in the 18 other districts.

4. The 2010 DHS rather than the more recent 2015 DHS was used to ensure that we capture characteristics before the start of the programme in 2014.

5. Only 320 of the 817 Crown Agents facilities are shown on the map, due to missing GIS data.
