# Supplementary material for: Who is paid in pay-for-performance? Inequalities in the distribution of financial bonuses amongst health centres in Zimbabwe
Source: Health Policy Plan. 2022 Jan 29;37(4):429–39. doi: 10.1093/heapol/czab154 (PMC9006063; doi:10.1093/heapol/czab154)
Supplement: czab154_Supp [file czab154_supp.zip › czab154_Supp/Appendix.docx]

**Appendix (Supplementary file – online only)**

## A1 – Bonus calculation

The following indicators were used to calculate the quantity bonus:

- 1. Curative care consultations OPD (new consultation)
  2. Four antenatal care visits
  3. Pregnant woman tested for HIV
  4. First ANC visit during the first 16 weeks of pregnancy
  5. ARV prophylaxis to HIV for pregnant woman
  6. Pregnant woman received TT2+
  7. Pregnant woman screened for syphilis
  8. Pregnant woman received Malaria prevention (2x IPT)
  9. Delivery attended by skilled health worker in health institution
  10. High-risk maternal cases referrals
  11. Two postnatal care visit
  12. First and repeat visits for short term family planning methods
  13. First and repeat visits for long term family planning methods
  14. Child aged one and under fully immunised (primary course)
  15. Vitamin A given to a child 6 to 59 months
  16. Growth Monitoring for children under five

The remoteness bonus captures five dimensions:

1. The distance of the rural health centre from its referral centre (district hospital)

2. The availability of communication (fixed telephone, mobile network) at the rural health centre

3. The condition of access roads to the rural health centre

4. The availability of public transport (for patients) to the rural health centre

5. The level (percentage) of the rural health centre catchment population living/residing more than 8km from the centre

The maximum amount that can be earned for remoteness if 30% of the quantity bonus. Remoteness was assessed the National Purchasing Agent and its local/decentralised unit- the Local Purchasing Unit in consultation with the District Health Executive.

## A2 – Desk review

We searched Google, Google Scholar, PubMed and Medline databases to identify published papers. We also searched grey literature obtained from the MoHCC along with reports and evaluations conducted by Cordaid, Crown Agents, the Global Fund, P4P stakeholders in Zimbabwe, the World Bank and local non-governmental organisations. A further search was conducted for any documentation pertaining to P4P bonus design, implementation and procedure manuals used by Cordaid, Crown Agents and the World Bank. MoHCC strategy documents (health plans) were downloaded from public websites. A time period from 2004 to January 2018 was defined in the initial search to narrow the searches to those documents most associated with P4P and results-based management programmes in Zimbabwe. Documents were systematically categorised, collated, read and analysed thematically by at least three members of the research team (Kadungure, Brown and Loewenson) with inter-rater agreement determined by consensus. Analysis inductively grouped interview responses into themes: the types of inequity perceived between facilities; explanations for why those inequities existed (pre-and post-P4P); facility and contextual characteristics affecting performance, perceptions of how P4P processes mitigated or enhanced identified inequities, and; the consequences on facility or personal performance. A total of 64 documents were analysed, including nine programme evaluations and review reports, twenty-six quarterly, biannual, counter-verification and programme financial reports, thirteen GoZ/MoHCC strategy/policy documents, four programme implementation manuals, eight published/journal reports on Zimbabwe, and eight P4P NSC meeting minutes.

## A3 – Total bonus and facility and local area characteristics

Table A1 – Log of total P4P bonus per quarter and facility characteristics at baseline (time-series linear regressions)

|  | (1) | (2) | (3) | (4) |
| --- | --- | --- | --- | --- |
| Proportion of clinical guidelines | 0.317** |  |  |  |
|  | (0.133) |  |  |  |
| Number of clinical staff |  | 0.045*** |  |  |
|  |  | (0.004) |  |  |
| Total 100 monthly consultations |  |  | 0.038*** |  |
|  |  |  | (0.003) |  |
| Drug availability index |  |  |  | 0.284 |
|  |  |  |  | (0.184) |
| Facilities | 87 | 87 | 87 | 87 |
| Observations (facility-quarters) | 852 | 852 | 852 | 852 |
| R-squared | 0.297 | 0.341 | 0.363 | 0.295 |

Notes: The outcome of interest in all models is the logged P4P bonus per facility per quarter. Results are from linear time-series regressions, including quarter, facility and province fixed effects. Robust standard errors in parentheses, *** p<0.01, ** p<0.05, * p<0.1

Table A2– Log of total P4P bonus and socio-economic characteristics of the local area (time-series linear regressions)

|  | (1) | (2) | (3) |
| --- | --- | --- | --- |
| **DHS wealth quintile:** |  |  |  |
| Second poorest wealth quintile | 1.151*** |  |  |
|  | (0.073) |  |  |
| Middle wealth quintile | 1.151*** |  |  |
|  | (0.069) |  |  |
| Second richest wealth quintile | 1.126*** |  |  |
|  | (0.067) |  |  |
| Richest wealth quintile | 1.035*** |  |  |
|  | (0.096) |  |  |
| **Remoteness of the facility:** |  |  |  |
| Second most remote quintile |  | 0.173*** |  |
|  |  | (0.048) |  |
| Middle remoteness quintile |  | 0.096*** |  |
|  |  | (0.026) |  |
| Second least remote quintile |  | 0.219*** |  |
|  |  | (0.064) |  |
| Least remote quintile |  | 0.085* |  |
|  |  | (0.043) |  |
| **Distance to district hospital:** |  |  |  |
| Second most remote quintile |  |  | 0.166*** |
|  |  |  | (0.021) |
| Middle remoteness quintile |  |  | -0.013 |
|  |  |  | (0.039) |
| Second least remote quintile |  |  | 0.309*** |
|  |  |  | (0.032) |
| Least remote quintile |  |  | -0.020 |
|  |  |  | (0.048) |
| Observations (facility-quarters) | 557 | 817 | 817 |
| R-squared | 0.431 | 0.398 | 0.344 |

Notes: The outcome of interest in all models is the logged P4P bonus per facility per quarter. Results are from linear time-series regressions, including quarter, facility and province fixed effects. Robust standard errors in parentheses, *** p<0.01, ** p<0.05, * p<0.1

Table A3– Log of total P4P bonus, facility characteristics and socio-economic characteristics of the local area (time-series linear regressions)

|  | (1) | (2) | (3) | (4) |
| --- | --- | --- | --- | --- |
| Proportion of clinical guidelines | 0.309* | 1.489*** | 0.962*** | 0.443** |
|  | (0.164) | (0.368) | (0.139) | (0.164) |
| Number of clinical staff | 0.024*** | 0.019* | 0.006 | 0.023*** |
|  | (0.004) | (0.010) | (0.007) | (0.004) |
| Total 100 monthly consultations | 0.030*** | 0.020*** | 0.023*** | 0.032*** |
|  | (0.003) | (0.003) | (0.002) | (0.002) |
| Drug availability index | -0.000 | -1.277*** | -0.419 | -0.002 |
|  | (0.243) | (0.313) | (0.229) | (0.240) |
| **DHS wealth quintile:** |  |  |  |  |
| Second poorest wealth quintile |  | 0.147 |  |  |
|  |  | (0.119) |  |  |
| Middle wealth quintile |  | 0.224** |  |  |
|  |  | (0.089) |  |  |
| Second richest wealth quintile |  | 0.445*** |  |  |
|  |  | (0.071) |  |  |
| Richest wealth quintile |  | - |  |  |
|  |  | - |  |  |
| **Remoteness of the facility:** |  |  |  |  |
| Second most remote quintile |  |  | 0.110* |  |
|  |  |  | (0.051) |  |
| Middle remoteness quintile |  |  | -0.056 |  |
|  |  |  | (0.076) |  |
| Second least remote quintile |  |  | -0.090 |  |
|  |  |  | (0.063) |  |
| Least remote quintile |  |  | 0.264** |  |
|  |  |  | (0.115) |  |
| **Distance to district hospital:** |  |  |  |  |
| Second most remote quintile |  |  |  | 0.020 |
|  |  |  |  | (0.053) |
| Middle remoteness quintile |  |  |  | 0.229*** |
|  |  |  |  | (0.038) |
| Second least remote quintile |  |  |  | 0.260*** |
|  |  |  |  | (0.030) |
| Least remote quintile |  |  |  | 0.118* |
|  |  |  |  | (0.056) |
| Observations (facility-quarters) | 852 | 319 | 519 | 792 |
| R-squared | 0.375 | 0.436 | 0.439 | 0.406 |

*Notes:* In Model 2, the richest wealth quintile is omitted due to collinearity.

## A4 – Results by bonus component

Table A4 – Quantity P4P bonus (logged) per quarter and facility characteristics at baseline

|  | (1) | (2) | (3) | (4) |
| --- | --- | --- | --- | --- |
| Proportion of clinical guidelines | 0.560*** |  |  |  |
|  | (0.107) |  |  |  |
| Number of clinical staff |  | 0.044*** |  |  |
|  |  | (0.003) |  |  |
| Total monthly consultations |  |  | 0.031*** |  |
|  |  |  | (0.002) |  |
| Drug availability index |  |  |  | -0.071 |
|  |  |  |  | (0.187) |
| Observations (facility-quarters) | 764 | 764 | 764 | 764 |
| R-squared | 0.382 | 0.416 | 0.415 | 0.374 |

Notes: The outcome of interest in all models is the logged P4P bonus per facility per quarter. Results are from linear time-series regressions, including quarter, facility and province fixed effects. Robust standard errors in parentheses, *** p<0.01, ** p<0.05, * p<0.1

Table A5 – Quantity bonus (logged) and socio-economic characteristics of the local area

|  | (1) | (2) | (3) |
| --- | --- | --- | --- |
| **DHS wealth quintile:** |  |  |  |
| Second poorest wealth quintile | 1.433*** |  |  |
|  | (0.191) |  |  |
| Middle wealth quintile | 1.410*** |  |  |
|  | (0.190) |  |  |
| Second richest wealth quintile | 1.350*** |  |  |
|  | (0.188) |  |  |
| Richest wealth quintile | 1.308*** |  |  |
|  | (0.169) |  |  |
| **Remoteness of the facility:** |  |  |  |
| Second most remote quintile |  | 0.156*** |  |
|  |  | (0.047) |  |
| Middle remoteness quintile |  | 0.049 |  |
|  |  | (0.038) |  |
| Second least remote quintile |  | 0.252*** |  |
|  |  | (0.062) |  |
| Least remote quintile |  | 0.163** |  |
|  |  | (0.057) |  |
| **Distance to district hospital:** |  |  |  |
| Second most remote quintile |  |  | 0.044** |
|  |  |  | (0.017) |
| Middle remoteness quintile |  |  | -0.072** |
|  |  |  | (0.028) |
| Second least remote quintile |  |  | 0.160*** |
|  |  |  | (0.036) |
| Least remote quintile |  |  | -0.223*** |
|  |  |  | (0.042) |
| Observations (facility-quarters) | 559 | 690 | 690 |
| R-squared | 0.444 | 0.478 | 0.420 |

Notes: The outcome of interest in all models is the logged P4P bonus per facility per quarter. Results are from linear time-series regressions, including quarter, facility and province fixed effects. Robust standard errors in parentheses, *** p<0.01, ** p<0.05, * p<0.1

Table A6 – Quality P4P bonus (logged) per quarter and facility characteristics at baseline

|  | (1) | (2) | (3) | (4) |
| --- | --- | --- | --- | --- |
| Proportion of clinical guidelines | 0.253** |  |  |  |
|  | (0.089) |  |  |  |
| Number of clinical staff |  | 0.048*** |  |  |
|  |  | (0.004) |  |  |
| Total monthly consultations |  |  | 0.042*** |  |
|  |  |  | (0.003) |  |
| Drug availability index |  |  |  | 0.485** |
|  |  |  |  | (0.181) |
| Observations (facility-quarters) | 752 | 752 | 752 | 752 |
| R-squared | 0.313 | 0.364 | 0.392 | 0.313 |

Notes: The outcome of interest in all models is the logged P4P bonus per facility per quarter. Results are from linear time-series regressions, including quarter, facility and province fixed effects. Robust standard errors in parentheses, *** p<0.01, ** p<0.05, * p<0.1

Table A7 – Quality bonus (logged) and socio-economic characteristics of the local area

|  | (1) | (2) | (3) |
| --- | --- | --- | --- |
| **DHS wealth quintile:** |  |  |  |
| Second poorest wealth quintile | 1.231*** |  |  |
|  | (0.085) |  |  |
| Middle wealth quintile | 1.254*** |  |  |
|  | (0.080) |  |  |
| Second richest wealth quintile | 1.173*** |  |  |
|  | (0.067) |  |  |
| Richest wealth quintile | 1.101*** |  |  |
|  | (0.107) |  |  |
| **Remoteness of the facility:** |  |  |  |
| Second most remote quintile |  | 0.141** |  |
|  |  | (0.047) |  |
| Middle remoteness quintile |  | 0.091*** |  |
|  |  | (0.027) |  |
| Second least remote quintile |  | 0.191** |  |
|  |  | (0.070) |  |
| Least remote quintile |  | 0.060 |  |
|  |  | (0.044) |  |
| **Distance to district hospital:** |  |  |  |
| Second most remote quintile |  |  | 0.176*** |
|  |  |  | (0.026) |
| Middle remoteness quintile |  |  | 0.000 |
|  |  |  | (0.048) |
| Second least remote quintile |  |  | 0.262*** |
|  |  |  | (0.040) |
| Least remote quintile |  |  | -0.044 |
|  |  |  | (0.061) |
| Observations (facility-quarters) | 496 | 730 | 730 |
| R-squared | 0.440 | 0.399 | 0.399 |

Notes: The outcome of interest in all models is the logged P4P bonus per facility per quarter. Results are from linear time-series regressions, including quarter, facility and province fixed effects. Robust standard errors in parentheses, *** p<0.01, ** p<0.05, * p<0.1

Table A8 – Remoteness supplement (logged) per quarter and facility characteristics at baseline

|  | (1) | (2) | (3) | (4) |
| --- | --- | --- | --- | --- |
| Proportion of clinical guidelines | -0.069 |  |  |  |
|  | (0.125) |  |  |  |
| Number of clinical staff |  | -0.006 |  |  |
|  |  | (0.005) |  |  |
| Total monthly consultations |  |  | -0.005 |  |
|  |  |  | (0.005) |  |
| Drug availability index |  |  |  | -0.736*** |
|  |  |  |  | (0.212) |
| Observations (facility-quarters) | 737 | 737 | 737 | 737 |
| R-squared | 0.299 | 0.299 | 0.299 | 0.302 |

Notes: The outcome of interest in all models is the logged P4P bonus per facility per quarter. Results are from linear time-series regressions, including quarter, facility and province fixed effects. Robust standard errors in parentheses, *** p<0.01, ** p<0.05, * p<0.1

Table A9 – Remoteness supplement (logged) and socio-economic characteristics of the local area

|  | (1) | (2) | (3) |
| --- | --- | --- | --- |
| **DHS wealth quintile:** |  |  |  |
| Second poorest wealth quintile | 0.039 |  |  |
|  | (0.133) |  |  |
| Middle wealth quintile | 0.257* |  |  |
|  | (0.131) |  |  |
| Second richest wealth quintile | 0.565*** |  |  |
|  | (0.123) |  |  |
| Richest wealth quintile | -0.094 |  |  |
|  | (0.207) |  |  |
| **Remoteness of the facility:** |  |  |  |
| Second most remote quintile |  | 0.229*** |  |
|  |  | (0.042) |  |
| Middle remoteness quintile |  | -0.087* |  |
|  |  | (0.039) |  |
| Second least remote quintile |  | -0.077 |  |
|  |  | (0.072) |  |
| Least remote quintile |  | 0.514*** |  |
|  |  | (0.036) |  |
| **Distance to district hospital:** |  |  |  |
| Second most remote quintile |  |  | 0.417*** |
|  |  |  | (0.040) |
| Middle remoteness quintile |  |  | 0.456*** |
|  |  |  | (0.060) |
| Second least remote quintile |  |  | 0.675*** |
|  |  |  | (0.044) |
| Least remote quintile |  |  | 0.594*** |
|  |  |  | (0.072) |
| Observations (facility-quarters) | 500 | 748 | 748 |
| R-squared | 0.313 | 0.346 | 0.360 |

Notes: The outcome of interest in all models is the logged P4P bonus per facility per quarter. Results are from linear time-series regressions, including quarter, facility and province fixed effects. Robust standard errors in parentheses, *** p<0.01, ** p<0.05, * p<0.1

## A5 – Time trends

Table A10 – Log of total P4P bonus and facility-characteristics, interactions by year

|  | (1) | (2) | (3) | (4) |
| --- | --- | --- | --- | --- |
| Year = 2015 | 1.584*** | 1.237*** | 1.205*** | 1.626*** |
|  | (0.354) | (0.094) | (0.107) | (0.410) |
| Year = 2016 | 2.011*** | 1.374*** | 1.468*** | 1.459*** |
|  | (0.441) | (0.208) | (0.207) | (0.509) |
| Proportion of clinical guidelines | 0.671 |  |  |  |
|  | (0.463) |  |  |  |
| 2015 * Guidelines | -0.389 |  |  |  |
|  | (0.411) |  |  |  |
| 2016 * Guidelines | -0.679 |  |  |  |
|  | (0.451) |  |  |  |
| Clinical staff (number) |  | 0.038 |  |  |
|  |  | (0.025) |  |  |
| 2015 * Clinical staff |  | 0.009 |  |  |
|  |  | (0.013) |  |  |
| 2016 * Clinical staff |  | 0.013 |  |  |
|  |  | (0.013) |  |  |
| Consultations (100 monthly) |  |  | 0.034** |  |
|  |  |  | (0.014) |  |
| 2015 * Consultations |  |  | 0.009 |  |
|  |  |  | (0.008) |  |
| 2016 * Consultations |  |  | 0.001 |  |
|  |  |  | (0.009) |  |
| Drug availability index |  |  |  | 0.544 |
|  |  |  |  | (0.956) |
| 2015 * Drug availability index |  |  |  | -0.596 |
|  |  |  |  | (0.617) |
| 2016 * Drug availability index |  |  |  | -0.059 |
|  |  |  |  | (0.714) |
| Observations | 852 | 852 | 852 | 852 |
| R-squared | 0.520 | 0.549 | 0.564 | 0.518 |

Notes: Robust standard errors in parentheses, *** p<0.01, ** p<0.05, * p<0.1

Table A11 – Log of total P4P bonus and facility-characteristics, interactions by year, controlling for facility and local-area characteristics

|  | (1) | (2) | (3) | (4) |
| --- | --- | --- | --- | --- |
| Year = 2015 | 2.064*** | 1.350*** | 1.320*** | 1.733** |
|  | (0.724) | (0.160) | (0.192) | (0.693) |
| Year = 2016 | 2.982*** | 1.934*** | 1.999*** | 1.993** |
|  | (0.441) | (0.208) | (0.207) | (0.509) |
| Proportion of clinical guidelines | 2.175** |  |  |  |
|  | (0.860) |  |  |  |
| 2015 * Guidelines | -0.806 |  |  |  |
|  | (0.864) |  |  |  |
| 2016 * Guidelines | -1.219 |  |  |  |
|  | (0.939) |  |  |  |
| Clinical staff (number) |  | 0.008 |  |  |
|  |  | (0.018) |  |  |
| 2015 * Clinical staff |  | 0.020 |  |  |
|  |  | (0.013) |  |  |
| 2016 * Clinical staff |  | 0.012 |  |  |
|  |  | (0.015) |  |  |
| Consultations (100 monthly) |  |  | 0.017 |  |
|  |  |  | (0.014) |  |
| 2015 * Consultations |  |  | 0.012 |  |
|  |  |  | (0.010) |  |
| 2016 * Consultations |  |  | -0.007 |  |
|  |  |  | (0.014) |  |
| Drug availability index |  |  |  | -1.065 |
|  |  |  |  | (1.336) |
| 2015 * Drug availability index |  |  |  | -0.511 |
|  |  |  |  | (0.985) |
| 2016 * Drug availability index |  |  |  | -0.024 |
|  |  |  |  | (1.002) |
| Observations | 319 | 319 | 319 | 319 |
| R-squared | 0.662 | 0.658 | 0.661 | 0.658 |

Notes: All models control for the socio-economic status of the local area, availability of clinical guidelines, number of staff, consultation volumes as well as availability of drugs. Robust standard errors in parentheses, *** p<0.01, ** p<0.05, * p<0.1

Table A12 – Log of total P4P bonus and socio-economic characteristics of the local area (time-series linear regressions)

|  | (1) | (2) | (3) |
| --- | --- | --- | --- |
|  |  |  |  |
| DHS wealth quintile = richest | 1.362** |  |  |
|  | (0.513) |  |  |
| Year = 2015 | 1.880** | 1.255*** | 1.188*** |
|  | (0.720) | (0.078) | (0.087) |
| Year = 2016 | 1.097 | 1.614*** | 1.248*** |
|  | (0.660) | (0.274) | (0.241) |
| 2015 * Richest quintile | -0.458 |  |  |
|  | (0.724) |  |  |
| 2016 * Richest quintile | -0.481 |  |  |
|  | (0.640) |  |  |
| Remoteness quintile = most remote |  | -0.016 |  |
|  |  | (0.231) |  |
| 2015 * Most remote |  | 0.194 |  |
|  |  | (0.141) |  |
| 2016 * Most remote |  | 0.076 |  |
|  |  | (0.149) |  |
| Distance to hospital = most remote |  |  | -0.130 |
|  |  |  | (0.212) |
| 2015 * Most remote to hospital |  |  | 0.104 |
|  |  |  | (0.128) |
| 2016 * Most remote to hospital |  |  | 0.250* |
|  |  |  | (0.133) |
| Observations | 557 | 817 | 792 |
| R-squared | 0.604 | 0.588 | 0.534 |

Notes: Only richest quintile and most remote quintile are shown. Time-trends for other quintiles are also non-significant. Robust standard errors in parentheses

*** p<0.01, ** p<0.05, * p<0.1

Table A13 – Log of total P4P bonus and socio-economic characteristics of the local area, controlling for facility characteristics (time-series linear regressions)

|  | (1) | (2) | (3) |
| --- | --- | --- | --- |
|  |  |  |  |
| DHS wealth quintile = richest | 1.430*** |  |  |
|  | (0.295) |  |  |
| Year = 2015 | 2.880*** | 1.299*** | 1.217*** |
|  | (0.078) | (0.084) | (0.101) |
| Year = 2016 | 2.894*** | 1.924*** | 1.356*** |
|  | (0.068) | (0.299) | (0.229) |
| 2015 * Richest quintile | -1.646 |  |  |
|  | (.) |  |  |
| 2016 * Richest quintile | -1.510*** |  |  |
|  | (0.034) |  |  |
| Remoteness quintile = least remote |  | -0.126 |  |
|  |  | (0.461) |  |
| 2015 * Least remote |  | 0.616* |  |
|  |  | (0.315) |  |
| 2016 * Least remote |  | 0.479 |  |
|  |  | (0.310) |  |
| Distance to hospital = least remote |  |  | -0.237 |
|  |  |  | (0.228) |
| 2015 * Least remote to hospital |  |  | 0.119 |
|  |  |  | (0.135) |
| 2016 * Least remote to hospital |  |  | 0.221 |
|  |  |  | (0.142) |
| Observations | 557 | 817 | 792 |
| R-squared | 0.604 | 0.588 | 0.602 |

Notes: Only richest quintile and most remote quintile are shown. Time-trends for other quintiles are also non-significant. All models control for availability of clinical guidelines, number of staff, consultation volumes as well as availability of drugs. Robust standard errors in parentheses

*** p<0.01, ** p<0.05, * p<0.1
